# Identification and management of patients at high-risk for cardiovascular disease in primary prevention

**DOI:** 10.1016/j.ajpc.2025.101265

**Published:** 2025-08-22

**Authors:** Michael Ahlers, Sonya John, Harpreet Bhatia, Viet Le, Pam Taub

**Affiliations:** a Division of Cardiovascular Medicine, University of California San Diego, La Jolla, CA, United States; b Cardiology Department, Intermountain Health, Canyons Region, Murray, UT, United States

**Keywords:** Coronary artery disease, Atherosclerosis, Primary care, Prevention, Preventive medicine, Cardiovascular risk factors

## Abstract

Atherosclerotic cardiovascular disease (ASCVD) is a complex pathophysiological process and encompasses a broad spectrum of disease, from subclinical atherosclerotic plaque to acute events with lifelong morbidity or sudden mortality. Many identified risk factors for ASCVD include elevated blood pressure, elevated total cholesterol, diabetes mellitus, overweight status, smoking, dietary factors, physical inactivity, and inadequate sleep.

The medical community has traditionally dichotomized patients into primary and secondary prevention, which may be too simplistic. Recent biomarker, imaging, and pharmacotherapy trials have highlighted the importance of recognizing and treating subclinical atherosclerosis to mitigate the progression of subclinical disease and reduce the risk for highly morbid or lethal ASCVD events. Here, we describe the patient with subclinical atherosclerosis and risk factors as a “high-risk primary prevention” patient. Employing an optimal therapeutic strategy over the lifetime of a high-risk primary prevention patient can mitigate ASCVD progression and prevent ASCVD events. This review highlights biomarkers for identifying high-risk primary prevention patients and reviews clinical trials in this population. We then describe current data that guide the management of these patients and discuss future directions for management.

## 1 Introduction

Historically, patients at risk for atherosclerotic cardiovascular disease (ASCVD) are categorized as primary (no prior history of clinical ASCVD) or secondary (clinical ASCVD) prevention. Much of the investigation related to ASCVD prevention is focused on secondary prevention, with relatively fewer seminal primary prevention trials, and more healthcare resources dedicated to secondary prevention. Over the past decade, however, there has been a shift in this dichotomous approach with mounting evidence identifying patients without clinical ASCVD but with high-risk features, not conforming to the traditional criteria for primary or secondary prevention. These patients are now termed "high-risk primary prevention," recognizing that they have multiple cardiovascular risk factors or evidence of subclinical disease**.**

**Fig. 1:**
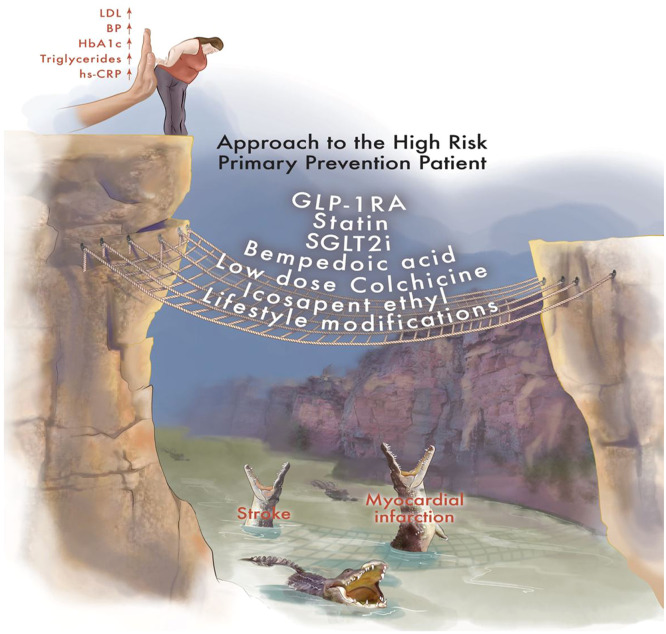
The High-Risk Primary Prevention Patient is defined by risk factors and subclinical ASCVD. A comprehensive therapeutic strategy can “catch” the patient from falling off the cliff and decrease risk of future ASCVD events.

Many of these patients are at very high risk with comorbidities such as diabetes, obesity, and elevated triglycerides. Some of these patients may have “minor” abnormalities when reviewed in isolation, such as abdominal obesity, mildly elevated fasting blood sugar, elevated blood pressure, and elevated triglycerides (see Fig. 1). However, many will meet the criteria for metabolic syndrome, which is associated with an increased risk of Type II diabetes and cardiovascular (CV) disease [1]. Metabolic syndrome is important to recognize and aggressively treat with lifestyle and pharmacotherapy.

High-risk primary prevention patients are often undertreated and have a very high ASCVD event rate compared to traditional primary prevention patients, though they are frequently categorized together. The academic community has started incorporating these patients into clinical trials and has learned how important it is to recognize and treat these patients early before they have a major ASCVD event. In this review, we will highlight how to identify these patients using biomarkers such as Lipoprotein(a) [Lp(a)], high sensitivity C-Reactive Protein (hs-CRP), and imaging tools such as coronary artery calcium (CAC) score. We will provide an overview of the major clinical trials in this population that have contributed to the evidence base to guide the management of these high-risk primary prevention patients. In addition, we review several ongoing clinical trials that will continue to provide insights into the optimal management of these patients. It is important to emphasize that the current evidence remains preliminary, and definitive clinical guidelines for routine management in these populations are still evolving, especially within the context of differing recommendations between US and European guidelines.

Clinicians should approach high-risk prevention patients holistically with comprehensive management strategies. The cornerstone of management for these patients is evidence-based lifestyle recommendations followed by pharmacotherapy. In addition, aggressive management of all known aspects of their cardiovascular risk should be pursued. Managing low-density lipoprotein cholesterol (LDL-C) and blood pressure alone is insufficient to prevent progression of ASCVD in many high-risk patients. Other crucial cardiovascular risk factors such as weight, dysglycemia, elevated triglycerides, elevated Lp(a), elevated inflammatory substrate, and renal dysfunction should be simultaneously evaluated and addressed. By doing so, we can improve patient outcomes and decrease long-term healthcare costs.

## 2 Tools to identify high-risk primary prevention patients

Many biomarkers are available for CV risk stratification, ranging from ceramides and myeloperoxidase to Lp(a) and hs-CRP. However, only some of these biomarkers and imaging modalities have significant clinical data recommending routine use. We will highlight biomarkers and imaging tools that have robust clinical data and should be considered for CV risk stratification.

### 2.1 Coronary calcium

Patients at high risk for ASCVD events include patients with advanced subclinical atherosclerosis. A powerful tool for identifying such patients is the quantification of CAC. In addition to dedicated screening with a CAC score, the utility of incidentally discovered calcification, such as in the aorta on computerized tomography (CT) scans of the chest obtained for other clinical purposes, or extra-coronary calcification such as aortic calcification or breast calcification, is increasingly recognized as helpful in predicting CV risk.

Current guidelines recommend consideration of CAC scoring in primary prevention for intermediate-risk patients when the treatment decision is uncertain after a quantitative risk assessment and shared decision-making (ACC/AHA IIb Recommendation, LOE B) [[2], [3], [4],^5^]. Even when statin therapy is already prescribed, CAC scoring may have benefit for therapeutic purposes, as guidelines recommend placing patients with CAC scores > 300 or ≥ the 75th percentile (normalized for age, sex, and ethnicity) into a higher risk category [2]. This may be actionable, including increasing therapy to a high-intensity statin [3].

CAC scoring may also help identify the highest-risk patients. In a recent study by Budoff et al. [6] 4949 patients from the CONFIRM registry with CAC scoring and long-term follow-up for subsequent major adverse cardiovascular events (MACE) were evaluated and categorized by baseline ASCVD status. The study population was 56 % male with a mean age of 57.6 years, and a similar burden of traditional cardiovascular risk factors as the general population. The primary endpoint was a MACE composite of all-cause mortality, nonfatal MI, and hospitalization for unstable angina (UA). Short-term revascularization within 90 days was excluded to minimize treatment bias induced by visualization of stenosis on incident coronary computed tomography angiography. Patients with CAC > 300 had a 20 % rate of MACE and 27 % rate of MACE or late revascularization over a median of 4.7 years of follow-up, which were similar to event rates of patients with prior ASCVD regardless of CAC score. Incidence of MACE increased with higher CAC score category in the patients with no prior ASCVD (4 % for CAC=0, 6 % for CAC 1–99, 11 % for CAC 100–299). These data suggest that patients with CAC > 300 have equivalent risk to patients with clinical ASCVD, and should be treated as such. The prevalence of increasing CAC score correlates strongly with age; greater than 25 % of white men aged 65–74 in the Multi-Ethnic Study of Atherosclerosis had a CAC score > 300 [7].

The timing of initial CAC scoring requires shared decision making between the patient and provider as well as weighting of each patient’s risk factors. In high-risk primary prevention patients, providers may consider recommending initial CAC testing in men after approximately age 35 and in women after age 48 for an approximately 25 % rate of detecting CAC score > 0 [8]. We do not recommend routine serial CAC testing in patients with an elevated CAC score, as there can be a paradoxical increase in calcium score with the initiation of statins and other lipid-lowering therapies, due to the positive remodeling of soft plaque to calcified plaque.

### 2.2 Lipoprotein(a) [Lp(a)]

Lipoprotein(a) [Lp(a)], an apolipoprotein B100-containing lipoprotein, is a well-established risk factor for atherosclerotic cardiovascular disease[[9], [10], [11], [12], [13]] and aortic stenosis [14,15] Lp(a) is produced by the liver, and has pro-inflammatory, atherogenic, and thrombotic effects [[9], [10], [11], [12], [13], [14], [15], [16]] Lp(a) levels are approximately 70 % to ≥90 % genetically determined, and median levels vary by ethnicity [[9], [10], [11], [12], [13]]. In the general population, the estimated prevalence of elevated Lp(a) is 20 % [17,18]. In a meta-analysis of 126,634 participants from 36 prospective studies, the relative risk of coronary heart disease (CHD) per 3.5-fold higher Lp(a) level adjusted for age and sex was 1.16 (95 % CI 1.11–1.22) [19]. This risk remained significant after adjustment for traditional risk factors, including systolic blood pressure, smoking, history of diabetes, and total cholesterol.

Mendelian randomization analyses demonstrated a linear relationship between absolute changes in Lp(a) level and risk of cardiovascular disease [9]. A 10 mg/dL lower genetically predicted Lp(a) concentration was associated with a 5.8 % lower CHD risk. In contrast, a 10 mg/dL lower genetically predicted LDL-C level estimated using an LDL-C genetic score was associated with a 14.5 % lower CHD risk [9]. In a study from MESA, in 4585 participants with 13.4 years of follow-up, elevated Lp(a) was associated with increased ASCVD risk regardless of baseline LDL-C [11].

There is currently a lack of consensus regarding Lp(a) risk thresholds; however, a threshold of 50 mg/dL or 75 nmol/L is most commonly cited [13]. The ACC/AHA guidelines have relative indications for lifetime screening, including a personal history of premature cardiovascular disease, family history of premature ASCVD (men, age <45 years; women, age <55 years), and family history of elevated Lp(a) and familial hypercholesterolemia. The European Society of Cardiology guidelines recommend obtaining an Lp(a) once in every individual’s lifetime, and most recently, the National Lipid Association has also recommended that Lp(a) be checked once in all adults [20].

Elevated Lp(a) is considered a risk-enhancing factor and, per the American Heart Association (AHA) and American College of Cardiology (ACC) guidelines, should prompt intensification of statin therapy and lower LDL-C goals than typically considered for a primary prevention patient. In addition, a risk-benefit discussion on aspirin should be considered in patients with elevated Lp(a) who have not had an ASCVD event. Recent data suggest that aspirin may be beneficial in the primary prevention setting for older patients with elevated Lp(a) genotypes [[21], [22], [23], [24]]. Although there is no current data to validate that lowering Lp(a) pharmacologically leads to a reduction in CV risk in primary or secondary risk groups, trials to address this question are ongoing. Multiple siRNA therapies to lower Lp(a) are in development. One siRNA, Lepodisiran, has included high-risk primary prevention patients in the enrolling ACCLAIM trial.

### 2.3 High sensitivity C-Reactive protein (hs-CRP)

Inflammation is critical to the pathogenesis and progression of atherosclerotic plaque, and high-sensitivity C-reactive protein (hs-CRP) is considered one of the best biomarkers for the identification and quantification of inflammation. Current guidelines recommend that hs-CRP quantification may be considered to inform therapy for primary prevention of ASCVD when the treatment decision is uncertain after quantitative risk assessment and shared decision making (ACC/AHA IIb Recommendation, LOE B), and guidelines informed by expert opinion recommend increasing estimated risk for patients with hs-CRP of ≥ 2 mg/L [2,25].

A meta-analysis conducted for the USPSTF, which aggregated data from 10 studies that adjusted for all seven Framingham risk factors, found a summary estimate of relative risk for incident coronary heart disease to be 1.58 (95 % CI, 1.37 to 1.83) for C-reactive protein (CRP) levels greater than 3.0 mg/L compared to levels less than 1.0 mg/L in patients without pre-existing cardiovascular disease [25]. Higher levels of hs-CRP are linked to an increased risk of incident coronary heart disease, regardless of whether comparing low (<1.0 mg/L) to average (1.0–3.0 mg/L) levels or average to high (>3.0 mg/L) levels [25]. However**,** routine use is not broadly recommended by bodies such as the USPSTF, emphasizing the preliminary status of evidence supporting routine clinical application**.**

In the Third National Health and Nutrition Examination Survey, the prevalence of high (> 3 mg/L) hs-CRP levels was significantly greater in patients with risk factors that increase risk of ASCVD including obesity (46.6 %), hypertension (38.8 %), diabetes (52.1 %), smoking history (27.2 %), female gender (31.0 %) or black ethnicity (34.9 %) (*P* < 0.001 for all compared to absence of characteristic) [26].

### 2.4 Inflammatory conditions (RA, SLE, psoriatic arthritis)

Systemic inflammation - as found in uncontrolled rheumatologic conditions including rheumatoid arthritis (RA), systemic lupus erythematosus (SLE), psoriatic arthritis, and inflammatory bowel disease - promotes atherosclerosis. and these conditions are known to have higher risk for ASCVD [27].

Patients with SLE have increased prevalence of ASCVD and disproportionately higher ASCVD event rates relative to their burden of traditional cardiovascular risk factors [[27], [28], [29], [30]]. Patients with RA also have elevated risk of ASCVD events and ASCVD-related mortality [27,31,32]. Rheumatoid arthritis is linked to an elevated risk of developing heart failure, independent of traditional cardiovascular risk factors such as coronary artery disease [33]. Additionally, higher levels of CRP are associated with an increased risk of incident heart failure [33]. Patients with psoriatic arthritis and inflammatory bowel disease also have increased risk of ASCVD in multiple studies [27,[34], [35], [36]]. Aggressive attenuation of inflammation likely mitigates the disproportionate risk of ASCVD in patients with these autoimmune conditions [27,33,37,38]. Chronic inflammatory disorders are included as a risk-enhancing factor in the latest ACC/AHA Guidelines of ASCVD risk assessment [2,27,39].

Emerging evidence indicates that elevated serum uric acid levels are independently associated with increased risk of ASCVD, even in individuals without traditional risk factors. The Kailuan study found a J-shaped association between SUA and cardiovascular events in a cohort of over 25,000 adults without known CVD, with increased risk observed above 5.0 mg/dL (300 μmol/L) [40]. The European Society of Cardiology acknowledges this association, but does not currently recommend urate-lowering therapy solely for cardiovascular prevention in asymptomatic individuals [41].

### 2.5 Estimated glomerular filtration rate (eGFR) and urine albumin-to-creatinine ratio (UACR)

Chronic kidney disease (CKD) is an underrecognized yet major risk factor for cardiovascular disease due to concomitant traditional risk factors such as type 2 DM and hypertension in addition to nontraditional risk factors including inflammation from overactivation of the renin-angiotensin-aldosterone system, oxidative stress, vascular complications, and abnormal calcium–phosphorus metabolism [42]. Studies have shown that glomerular filtration rate (GFR) below 60 ml/min/1.73 m^2^ and urine albumin-to-creatinine ratio (UACR) of 1.1 mg/mmol are independent predictors of all-cause mortality and cardiovascular mortality. With GFR < 60, the probability of developing coronary artery disease increases linearly, and patients with CKD stages G3a to G4 (15–60 ml/min/1.73 m^2^) have approximately double to triple the CVD mortality risk, respectively, relative to patients without CKD [43]. CKD has also been shown to be an independent risk factor for patients presenting with acute coronary syndrome compared to patients with stable exertional coronary disease [44]. While routine asymptomatic screening has not been established, aggressive measures should be taken to modify risk factors. Although no specific lipid targets are established in CKD patients, they should be treated similar to other high risk populations. The Study of Heart and Renal Protection (SHARP) trial [45] randomized 9270 participants with CKD without known coronary artery disease to a combination of simvastatin and ezetimibe or placebo. The primary composite outcome of nonfatal MI, cardiac death, stroke, or arterial revascularization was significantly reduced in the intervention arm compared to the placebo group with a relative risk reduction of 0.83. For patients with hypertension and CKD with or without diabetes, renin-angiotensin-aldosterone inhibitors are first line agents. For patients with concomitant diabetes, SGLT-2 inhibitors and finerenone are recommended for prevention of cardiovascular disease progression [46,47]. In CANVAS, approximately 34 % of the cohort was enrolled for primary prevention. While overall event rates were lower in the primary prevention cohort, canaglifozin reduced the primary endpoint compared to placebo (hazard ratio [HR] 0.86; 95 % confidence interval [CI], 0.75–0.97; *P* < 0.001 for noninferiority, *P* = 0.02) [48]. Similar reductions in MACE were seen in the canaglifozin cohort in the CREDENCE trial [49]. The FIDELIO-DKD and FIGARO-DKD trials demonstrated that finerenone significantly reduced renal and cardiovascular outcomes in patients with type 2 diabetes mellitus and a broad spectrum of CKD. The pooled analysis (FIDELITY) confirmed a benefit in cardiovascular outcomes with finerenone irrespective of ASCVD history [50].

### 2.6 Ankle brachial index

The ankle-brachial index (ABI), the ratio of systolic blood pressure in the ankle to that of the arm, is a simple maneuver for detecting peripheral artery disease that has been in use for decades. A low ABI, diagnostic for PAD, is strongly predictive of cardiovascular disease and overall mortality among both men and women without known CVD [51,52]. A meta-analysis of 16 population cohort studies demonstrated that the 10-year cardiovascular mortality in men with a low ABI (≤ 0.90) was 18.7 % (13.3 % to 24.1 %) and with normal ankle brachial index (1.11 to 1.40) was 4.4 % (3.2 % to 5.7 %). Corresponding mortalities in women were 12.6 % (6.2 % to 19.0 %) in the low ABI group and 4.1 % (2.2 % to 6.1 %) in the normal ABI group. It provided further risk stratification within each Framingham risk category for 10-year total mortality, cardiovascular mortality, and major coronary event rate for patients without known coronary artery disease [53]. Considered a tool to measure subclinical atherosclerosis, ABI has been established as a risk enhancer in the 2014 AHA/ACC guidelines on the primary prevention of atherosclerotic cardiovascular disease [2]. For patients with borderline ASCVD risk, ABI can help with risk stratification and affect the threshold for statin initiation or intensification [13]. Ankle-brachial index testing is recommended in asymptomatic patients with self-limited activity or functional impairment comparable to symptomatic claudication, as well as patients with claudication symptoms [54].

## 3 Review of clinical trials with high-risk primary prevention patients

Most contemporary clinical trials have focused on patients with established ASCVD. However, there is an emerging body of data in which high-risk primary prevention patients were enrolled that is providing insights on how to manage these patients (Table 1). These trials not only evaluate the efficacy of lipid-lowering therapies, such as statins and novel agents, but also explore interventions targeting other risk-enhancing conditions, including diabetes, CKD, and inflammation. These studies help to refine preventive strategies and expand the therapeutic toolkit for clinicians managing high-risk populations.

**Table 1:** Modern cardiovascular clinical outcomes trials including patients at high risk for primary ASCVD events.

Trial	Number of primary prevention patients; Number of Patients	Treatment	High Risk Primary Prevention Patient Definition	Primary Outcome	Primary Outcome Result	High Risk Primary Prevention Subanalysis Results
**JUPITER**	17,802; 17,802	Rosuvastatin 20 mg daily	Men: age greater than 50 years; Women: age greater than 60. CRP > 2 mg/L	Occurrence of first CV event: nonfatal myocardial infarction, nonfatal stroke, hospital- ization for unstable angina, an arterial revascularization procedure, or confirmed death from cardiovascular causes	Event rate 0.77/100 person-year in the rosuvastatin group compared to 1.36 in placebo; HR 0.56 (*p* < 0.0001). 50 % decrease in LDL-C, 37 % decrease in hs-CRP	**n/a**
**MEGA**	3966; 3966	pravastatin 10–20 mg/day	**n/a**	Occurrence of CHD (nonfatal and fatal MI, sudden cardiac death, UA, coronary revascularization)	33 % relative reduction in CHD events in the pravastatin arm vs control (66 events vs 101 events; HR 0.67, 95 % CI: 0.49–0.91, *P* = 0.01)	**n/a**
**CLEAR Outcomes**	4206; 13,970	Bempedoic acid 180 mg daily	any of the following: 1) Reynolds Risk score of > 30 % or a SCORE Risk score > 7.5 % over 10 years. 2) A CAC score > 400 Agatston units. 3) A patient with diabetes mellitus aged greater than 65 years for women or 60 years for men	Composite of death from cardiovascular causes, nonfatal myocardial infarction, nonfatal stroke, coronary revascularization	Event rate 11.7 % with bempedoic acid and 13.3 % with placebo. HR 0.87, 95 % CI 0.79 to 0.96, *P* = 0.004	Event rate 5.3 % in the bempedoic acid group and 7.6 % in the placebo group. HR 0.70, 95 % CI 0.55 to 0.89, *P* = 0.002
**THEMIS**	19,220; 19,220	Ticagrelor 90 twice daily, 60 mg twice daily	Age greater than 50 years with history of stable coronary artery disease (history of PCI, CABG, or stenosis of at least 50 %) and type 2 diabetes mellitus	Composite of cardiovascular death, myocardial infarction, or stroke	Primary outcome occurred in 7.7 % of patients int he ticagrelor group compared to 8.5 % in the placebo group	**n/a**
**REDUCE-IT**	2394; 8179	Icosapent ethyl 4 g daily	Age greater than 50 years with a history of diabetes mellitus and at least 1 additional risk factor for cardiovascular disease	Composite of 1) death from cardiovascular causes 2) nonfatal myocardial infarction 3) nonfatal stroke 4) coronary revascularization 5) unstable angina requiring hospitalization	Event rate 17.2 % with icosapent ethyl and 22.0 % with placebo. HR 0.75, 95 % CI 0.68–0.83, *P* < 0.001	Event rate of 12.2 % with icosapent ethyl and 13.6 % with placebo. HR 0.88, 95 % CI 0.70–1.10
**SPIRE Trials**	4253; 27,438	Bococizumab 150 mg every two weeks	male patients at least 50 years old and female patients at least 60 years old if they had either a history of diabetes, chronic kidney disease, or peripheral vascular disease and an additional risk factor, or familial hypercholesterolemia	nonfatal myocardial infarction, nonfatal stroke, hospitalization for unstable angina requiring urgent revascularization, or cardiovascular death	SPIRE-2 event rate 3.32 with bococizumab and 4.19 with placebo per 100 patient-years. HR 0.79, 95 % CI 0.65–0.97, *P* = 0.02	**unpublished**
**REWIND**	6787; 9901	Dulaglutide 1.5 mg weekly	At least 50 years old, Type 2 DM; In primary prevention had to have a least two of the following: tobacco use, dyslipidemia, hypertension, or abdominal obesity	First occurrence of the composite endpoint of non-fatal myocardial infarction, non-fatal stroke, or death from cardiovascular causes (including unknown causes)	Incidence rate of 2.4 per 100 person-years in the dulaglutide group 2.7 per 100 person-years in the placebo group (*p* = 0·026). All-cause mortality did not differ between groups	HR on the primary outcome was similar in participants with and without previous cardiovascular disease (*p* = 0.97)
**CANVAS Trials**	3489; 10,142	Canagliflozin 100 mg or 300 mg daily	at least 50 years of age with at least two of the following risk factors: diabetes for at least 10 years, systolic blood pressure > than 140 mm Hg while on at least one antihypertensive agent, current smoking, albuminuria, and high-density lipoprotein of less than 38.7 mg/dL	composite of death from cardiovascular causes, nonfatal myocardial infarction, or nonfatal stroke	Event rate 26.9 with canagliflozin and 31.5 with placebo per 1000 patient-years. HR 0.86, 95 % CI 0.75 to 0.97, *P* < 0.001 for noninferiority, *P* = 0.02 for superiority	Event rate 15.8 with canagliflozin and 15.5 with placebo per 1000 patient-years. HR 0.98, 95 % CI 0.74–1.30, *P* = 0.18
**LoDoCo2**	884; 5522	Colchicine 0.5 mg daily	CAD defined by coronary angiography or CAC score > 400.	Composite of cardiovascular death, myocardial infarction, ischemic stroke, or ischemia-driven coronary revascularization	Event rate 2.5 with colchicine and 3.6 with placebo per 100 person-years. HR 0.69, 95 % CI 0.57–0.83, *P* < 0.001	No prior ACS HR 0.81, 95 % CI (0.52–1.27). No prior coronary revascularization HR 0.72, 95 % CI 0.46–1.12

*g* = gram, mg = milligram, dL = deciliter, HR = Hazard Ratio, CI = Confidence Interval, CAD = coronary artery disease, CAC = coronary artery calcium.

### 3.1 Lipid lowering therapy- statins

#### 3.1.1 JUPITER trial

The Justification for the Use of Statins in Prevention: An Intervention Trial Evaluating Rosuvastatin (JUPITER) trial [55] randomized 17,802 non-diabetic men and women (38.2 %) without cardiovascular disease, LDL-*C* < 130 mg/dL, and hs-CRP ≥ 2.0 mg/L to rosuvastatin 20 mg/day or placebo. This trial was conceived on the basis that half of strokes and MIs occur in patients with LDL-C and total cholesterol within the defined normal range and that measurement of high-sensitivity CRP as a biomarker independently predicted future vascular events. With a median follow up time of 1.9 years, there was a 50 % decrease in LDL-C, a 37 % decrease in hs-CRP, and a 44 % decrease in the composite primary endpoint of myocardial infarction (MI), stroke, arterial revascularization, hospitalization for unstable angina, or death from cardiovascular causes. The 4-year number needed to treat (NNT) to prevent 1 primary endpoint was 31. This was a landmark clinical trial that demonstrated high event rate in patients with “normal” LDL-C levels and that additional biomarkers are needed for cardiovascular risk stratification.

#### 3.1.2 MEGA trial

The Primary Prevention of Cardiovascular Disease with Pravastatin in Japan (MEGA) trial [56] was a prospective, randomized, blinded study used to evaluate the benefit of statins in an Asian population with overall low risk for cardiovascular disease. The study enrolled 3966 participants to a heart healthy diet and 3866 participants to pravastatin 10 to 20 mg/day and the same diet recommendations. (68 % of the total study population were women). The primary endpoint for the study was the first occurrence of coronary heart disease (nonfatal and fatal MI, sudden cardiac death, UA, coronary revascularization). After an average follow-up of 5.3 years, there was a 33 % relative reduction of the primary endpoint in the pravastatin arm versus the control (HR 0.67, 95 % CI: 0.49–0.91, *P* = 0.01). The NNT at 5.3 years to prevent 1 primary endpoint event was 119. At the end of the mean follow up period there was a significant reduction in LDL-C concentration in the diet plus pravastatin group compared with the diet group (–18 % (59.8 mg/dL) vs –3 (70.2 mg/dL)%, *p* < 0·0001). This study demonstrated that in a relatively low risk population, primary prevention with low-dose statin therapy can be effective in reducing cardiac events even with a moderate reduction in LDL-C levels.

### 3.2 Lipid lowering Therapy- non-statins

#### 3.2.1 CLEAR outcomes

The CLEAR Outcomes trial investigated the effects of bempedoic acid versus placebo on cardiovascular outcomes in statin-intolerant patients with or at high risk for cardiovascular disease. This randomized, double-blind study included patients unable or unwilling to take greater than an average daily dose above the lowest approved dose of a statin. Of the participants, 30.1 % had no prior cardiovascular events. High risk for a primary cardiovascular event was defined by at least one of the following criteria: (1) a Reynolds Risk score above 30 % or a SCORE Risk score over 7.5 % over 10 years, (2) a coronary artery calcium score exceeding 400 Agatston units at any time in the past, or (3) diabetes in patients aged over 65 years for women or over 60 years for men. Inclusion required fasting LDL-C levels above 100 mg/dL while on optimized LDL-C-lowering therapy. A total of 13,970 patients were randomized for a median follow-up of 40.6 months, with a mean baseline LDL-C of 139.0 mg/dL, median triglycerides of 159.0 mg/dL, and median hs-CRP of 2.3 mg/L [57].

The primary endpoint was a 4-point MACE composed of 1) death from cardiovascular causes 2) nonfatal myocardial infarction 3) nonfatal stroke 4) coronary revascularization. The primary end point rate was 11.7 % in the bempedoic acid group and 13.3 % in the placebo group (819 patients [11.7 %] vs. 927 [13.3 %]; hazard ratio, 0.87; 95 % CI: 0.79 to 0.96; *P* = 0.004). Bempedoic acid significantly lowered LDL-C level (mean decrease over baseline of −21.1 % [95 % CI −21.9 % to −20.3 %] at 6 months) and hs-CRP (decrease over baseline of −21.6 % [95 % CI −23.7 % to −19.6 %] at 6 months) compared to placebo.

In prespecified analysis of the 4206 patients who were high risk for cardiovascular risk but without a primary event, baseline characteristics were similar except for older age (mean years 67.9 vs. 65.5) and higher prevalence of diabetes mellitus (66.1 % vs. 45.7 %) in the high risk primary prevention group compared to secondary prevention patients [58]. The primary 4-component composite end point rate was 5.3 % in the bempedoic acid group and 7.6 % in the placebo group (111 patients vs. 161; HR 0.68 [95 % CI, 0.53 to 0.87] [58]. Similar reductions in LDL (−21.3 %) and hs-CRP (−21.5 %) were observed with bempedoic acid use in this high risk primary prevention cohort compared to overall population [58].

#### 3.2.2 REDUCE-IT trial

The Reduction of Cardiovascular Events with Icosapent Ethyl-Interventional Trial (REDUCE-IT) was a randomized, double-blind, icosapent ethyl versus placebo trial for patients at high risk for cardiovascular events [59,60]. Inclusion criteria included either hypertriglyceridemia (135 to 499 mg/dL) with established cardiovascular disease, or history of diabetes mellitus aged greater than 50 years with at least 1 risk factor for cardiovascular disease. In this trial, LDL-C was required to be controlled at baseline to a level of 41 to 100 mg/dL and with stable statin use. The study randomized 8179 patients with a median follow up of 4.9 years. The median baseline LDL-C was 75 mg/dL. The median triglyceride level was 216.0 mg/dL. 29.3 % had not had a prior cardiovascular event.

The primary endpoint was a 5-point MACE consisting of 1) death from cardiovascular causes 2) nonfatal myocardial infarction 3) nonfatal stroke 4) coronary revascularization 5) unstable angina requiring hospitalization. Patients treated with 4 g/day of icosapent ethyl had a 4.8 % absolute risk reduction (22.0 % event rate without compared to 17.2 % with icosapent ethyl) and 24.8 % relative risk reduction in the composite endpoint (HR 0.75 (95 % CI 0.68–0.83, *P* < 0.001). The primary-prevention cohort of the study had a composite event rate of 13.6 % compared to 12.2 % with use of icosapent ethyl, which trended toward a reduction of events that was not statistically significant (163 patients (13.6 %) vs. 146 patients (12.2 %), HR 0.88 [95 % CI 0.70–1.10]). The median change in triglyceride level from baseline to 1 year was a decrease of 19.7 % greater with the use of icosapent ethyl compared to placebo (*P* < 0.001). Baseline triglyceride levels of normal or achievement of normal triglyceride levels at 1 year follow up did not significantly change the composite outcomes, suggesting pleiotropic benefits beyond triglyceride lowering. hs-CRP measured significantly lower at two years of follow up with use of icosapent ethyl compared to placebo despite similar baseline levels of hs-CRP in the two groups.

#### 3.2.3 SPIRE-1 and SPIRE-2 trials

The SPIRE Trials [61] evaluated the effects of proprotein convertase subtilisin-kexin type 9 (PCSK9) inhibition with the monoclonal antibody bococizumab on future adverse cardiovascular events. Combined analysis of these randomized, double blinded, placebo-controlled trials of 27,438 patients who received 150 mg of subcutaneous bococizumab every two weeks evaluated a primary endpoint of nonfatal myocardial infarction, nonfatal stroke, hospitalization for unstable angina requiring urgent revascularization, or cardiovascular death over a median follow-up of 10 months.

Patients were eligible for enrollment if they had a previous cardiovascular event. In addition, there was a high risk primary prevention cohort that consisted of male patients at least 50 years old and female patients at least 60 years old if they had either a history of diabetes, CKD, or peripheral vascular disease and also one of the following risk factors: asymptomatic coronary stenosis on cardiac imaging, smoking history, HDL < 40 mg/dL, hs-CRP > 2.0 mg/L, Lp(a) of > 50 mg/dL, microalbuminuria, or history of familial hypercholesterolemia (age cutoff decreased at least 35 years for men and 45 years for women with FH).

Baseline characteristics of the 27,438 patients included mean age 62.9 years old, 29.6 % female, 84.5 % high-intensity statin use, and 15.5 % meeting the high-risk primary prevention characteristics. Mean LDL-C was 93.8 mg/dL in SPIRE-1 and 133.6 mg/dL in SPIRE-2. Median hs-CRP was 2.0 mg/L. At 14 weeks, patients in the bococizumab cohort had a mean 56 % reduction in LDL-C compared to baseline, as well as significant (*P* < 0.001 for all) reductions in apolipoprotein B (55.3 %), Lp(a) (30.9 %) and triglycerides (19.4 %). Baseline hs-CRP was not significantly changed. The primary composite endpoint of cardiovascular death, nonfatal myocardial infarction, nonfatal stroke, hospitalization for unstable angina requiring revascularization occurred significantly less often in the group treated with bococizumab in SPIRE-2, which had a higher baseline LDL-C (occurring 3.32 versus 4.19 per 100 patient-years; HR 0.79, 95 % CI 0.65–0.97; RRR 21 %, *P* = 0.02), but was not statistically significant in SPIRE-1 at only 7 months median follow up. The patients with familial hypercholesterolemia treated with bococizumab had similar reduction in the primary outcome compared to patients with similar lipid profiles (HR 0.83, 95 % CI 0.44–1.54, *P* = 0.55) [62]. Development of bococizumab as a therapy was discontinued due to observed development of antidrug antibodies, but multiple other fully human PCSK9-inhibitor monoclonal antibodies remain available for prescription. Outcome analysis of only the high-risk primary prevention cohort has not been published separately.

### 3.3 Other therapeutic agents

#### 3.3.1 THEMIS trial

Ticagrelor, a reversible antagonist of the platelet P2Y12 receptor, has been shown to be beneficial in acute myocardial infarctions as well as high-risk patients with extensive coronary disease. The effect of ticagrelor on health outcomes in diabetes mellitus patients intervention study (THEMIS) [63]. Was a randomized, double-blind study, which evaluated ticagrelor plus aspirin versus aspirin alone in patients with stable coronary artery disease and type II diabetes, excluding those with prior MI or stroke. Among 19,220 patients followed for a median of 39.9 months, ticagrelor reduced ischemic cardiovascular events (7.7 % vs. 8.5 %, HR 0.90, *P* = 0.04), primarily due to fewer MIs and strokes. However, major bleeding and intracranial hemorrhage were significantly higher in the ticagrelor group (2.2 % vs. 1.0 %, HR 2.32, *P* < 0.001). The number needed to harm for a period of 36 months was 93 in the modified intention-to-treat population. Weighing both efficacy and safety events, adding ticagrelor did not reduce the risk of a composite outcome of net irreversible harm suggesting that ticagrelor in this patient population did not have a favorable risk-benefit ratio. Despite this, in 2020 the Food and Drug Administration expanded the indication for ticagrelor to primary prevention for patients who are at high risk for acute coronary syndrome and stroke.

#### 3.3.2 REWIND trial

Initial data showed glucagon-like-peptide-1 receptor agonists (GLP-1 RA) significantly reduced the risk of MACE in patients with type 2 diabetes. There have been at least five trials that assessed the cardiovascular effects of this class agent; lixisenatide (ELIXA; *n* = 6068)[64], albiglutide (Harmony Outcomes; *n* = 9463)[65], liraglutide (LEADER; *n* = 9340)[66], semaglutide (SUSTAIN-6; *n* = 3297)[67], or long-acting exenatide (EXSCEL; *n* = 14 752)[68] versus placebo on incident cardiovascular outcomes in people with type 2 diabetes. These trials included a high proportion of people with established atherosclerotic cardiovascular disease.

The REWIND trial [69,70], published in 2019 (*n* = 9901), enrolled a low proportion of patients with existing cardiovascular disease (31.5 %) and a higher proportion of women (46.3 %) compared to its preceding trials. This was a multicenter, randomized, double-blind, placebo-controlled trial that randomly assigned (1:1) either weekly subcutaneous injection of dulaglutide (1.5 mg) or placebo. Inclusion criteria included patients aged 50 or older with established or newly detected type 2 diabetes with a Hba1C less than 9.5 %. Patients aged 50 or older had to have established diagnosis of vascular disease (ie, a previous myocardial infarction, ischemic stroke, revascularization, hospital admission for unstable angina, or imaging evidence of myocardial ischemia) and those aged 55 years or older had to have vascular stenosis exceeding 50 % or left ventricular hypertrophy. Patients who were older than 60 had to have at least two risk factors for cardiovascular disease. The primary outcome was the first occurrence of any component of the composite outcomes which included nonfatal MI, nonfatal stroke, and death from either cardiovascular or unknown causes. The primary outcome occurred in 594 (12 %) assigned to dulaglutide and 663 (13.4 %) assigned to placebo (HR 0.88, 95 % CI 0.79–0.99; *p* = 0.026). Within subgroups the hazard ratio of the intervention on the primary outcome was similar in participants with and without previous cardiovascular disease suggesting that dulaglutide might be effective for both primary and secondary cardiovascular prevention in a high proportion of people with type 2 diabetes. Due to these promising results, the FDA approved the use of dulaglutide for ASCVD primary prevention in patients with type 2 diabetes, the only agent in its class with this indication.

#### 3.3.3 CANVAS and canvas-r trials

Although initially used to control type 2 diabetes, sodium-glucose cotransporter (SGLT-2) inhibitors have also been proven to improve cardiovascular outcomes in recent trials. The mechanism of action is thought to be through pleiotropic effects including natriuresis, diuresis, and blood pressure reduction. CANVAS and CANVAS-R[48] were two randomized, double-blinded, placebo-controlled, international trials reporting the effects of canagliflozin on cardiovascular outcomes in patients with type 2 diabetes (Hba1c ≥ 7.0 % and ≤ 10.5 %) and at high risk for adverse cardiovascular events.

The primary outcome was a composite measure of cardiovascular death, nonfatal myocardial infarction, or nonfatal stroke, assessed in 10,142 patients over an average follow-up period of 3.6 years. Of these participants, 34.4 % were enrolled due to a high risk for a primary cardiovascular event, defined as age 50 or older with at least two of the following risk factors: diabetes for over 10 years, systolic blood pressure above 140 mm Hg while on antihypertensive therapy, current smoking, albuminuria, or an HDL below 38.7 mg/dL. Participants were required to have a baseline eGFR of at least 30 ml/min/1.73 m². The mean baseline characteristics included an age of 63.3 years, 35.8 % female, and a diabetes duration of 13.5 years, with a mean HbA1c of 8.2 %.

The primary outcome rate was lower with canagliflozin than with placebo, occurring in 26.9 vs. 31.5 participants per 1000 patient-years (HR 0.86; 95 % CI, 0.75–0.97; *P* < 0.001 for noninferiority; *P* = 0.02 for superiority). Although each component of the composite outcome showed a trend toward benefit with canagliflozin, none reached statistical significance individually. Hospitalization for heart failure was significantly reduced with canagliflozin compared to placebo, occurring in 5.5 vs. 8.7 participants per 1000 patient-years (HR 0.67; 95 % CI, 0.52–0.87). In subgroup analysis, the benefit of the composite outcome was observed only in patients with baseline symptomatic ASCVD and was not seen in the high-risk primary prevention group (15.8 vs. 15.5 participants per 1000 patient-years; HR 0.98; 95 % CI, 0.74–1.30). Premature discontinuation of canagliflozin in 29.2 % of patients may have weakened the primary outcome effect.

#### 3.3.4 LoDoCo2 trial

The LoDoCo2 Trial [71] aimed to assess the effect of colchicine on the risk of ASCVD events in patients with chronic coronary artery disease. Although the majority of the 5522 patients included in the randomized, placebo-controlled, double-blinded LoDoCo2 Trial had a history of acute coronary syndrome at least six months before enrollment, 16 % were of high-risk primary prevention for acute coronary syndrome, defined as having coronary artery disease based on angiography or CAC score greater than 400 [71] Treatment with colchicine 0.5 mg daily was associated with a significant reduction in a composite of MACE including cardiovascular death, myocardial infarction, ischemic stroke, or ischemia-driven coronary revascularization (6.8 % in colchicine group vs 9.6 % in the placebo group; 2.5 vs. 3.6 events per 100 person-years; HR 0.69, 95 % CI 0.57–0.83; *P* < 0.001) over a median follow up of 28.6 months. The subgroups of no prior acute coronary syndrome and no prior coronary revascularization each had a numerically lower number of primary endpoint events in the colchicine group, which were not statistically significant (Table 1).

### 3.5 Ongoing clinical trials

#### 3.5.1 Victorian-1 prevent

Inclisiran is a novel short interfering RNA (siRNA) that inhibits the translation of the protein PCSK-9 in hepatocytes. It is given subcutaneously on day 1, followed by a second injection on day 90, and then an additional injection every 6 months. Based on the results from the phase-2 ORION-1 trial [72], the two-dose 300-mg inclisiran regimen produced the greatest reduction in LDL-C levels with a mean reduction in LDL-C levels from baseline at day 240 ranging between 26.7–47.2 % [72]. This robust reduction in LDL-C was also seen in ORION-3, the 4-year open-label extension study of ORION-1 [73]. In the phase-3 ORION-9 trial, the efficacy of inclisiran was evaluated in patients with heterozygous familial hypercholesterolemia on a background of maximally tolerated statin therapy ± ezetimibe with a baseline LDL-*C* ≥ 100 mg/dL (25 % of patient had preexisting coronary artery disease). At the end of the study period, there was an average LDL-C reduction of 47.9 % (95 % CI −53.5 to −42.73; *P* < 0.001) when compared to placebo [74]. A subsequent trial, ORION-11 evaluated the efficacy of inclisiran in patients with established atherosclerotic cardiovascular disease or an atherosclerotic cardiovascular disease risk equivalent (diabetes mellitus, familial hypercholesterolemia, or 10-year ASCVD risk ≥20 % as assessed by the Framingham Risk Score for Cardiovascular Disease). It demonstrated a similar LDL-C reduction, with a placebo-corrected reduction of 49.9 % (95 % CI −53.1 to −46.6; *P* < 0.001) with similar results seen in established cardiovascular disease or risk equivalent [75]. Although there are no completed cardiovascular outcomes trials for inclisiran, an exciting ongoing trial VICTORIAN-1 PREVENT [76]. is evaluating a potential role of inclisiran in MACE reduction among high-risk primary prevention patients such as those with elevated coronary artery calcium scores or non-obstructive coronary plaque seen on a coronary CT. In this event-driven trial, the primary outcome is time to composite endpoint of cardiovascular death, non-fatal myocardial infarction, non-fatal ischemic stroke, and urgent coronary revascularization. Results from this trial will elucidate the role for inclisiran in this high-risk population and help determine the most appropriate patient profile for this therapeutic.

#### 3.5.2 VESALIUS

The Effect of EVolocumab in PatiEntS at High CArdiovascuLar RIsk WithoUt Prior Myocardial Infarction or Stroke (VESALIUS)-CV trial [77] is a randomized, double-blind, placebo-controlled trial evaluating the effect of evolocumab in patients with high cardiovascular risk but without a previous ischemic event. The trial randomized 12,301 patients in a 1:1 ratio to receive either evolocumab 140 mg every 2 weeks or a matching placebo. Patients in this primary prevention cohort had atherosclerosis or high-risk diabetes. They also had an LDL-*C* ≥ 90 mg/dL (≥2.3 mmol/L), non-HDL-*C* ≥ 120 mg/dL (≥3.1 mmol/L), or apolipoprotein *B* ≥ 80 mg/dL (≥1.56 µmol/L) on at least 2 weeks of stable, optimized lipid-lowering therapy. The primary efficacy objective was to assess the risk of separate composite outcomes of coronary heart disease, death, MI, ischemic stroke, or ischemia driven arterial vascularization. This trial is first in its kind to evaluate the efficacy of a PCSK-9 inhibitor in primary prevention and should provide guidance of efficacy and safety of evolocumab in this cohort.

#### 3.5.3 CORALreef outcomes

The ongoing CORALreef Outcomes trial [78] is a phase 3, randomized, placebo-controlled study of the efficacy and safety of enlicitide chloride (MK-0616), an oral PCSK-9 inhibitor, in patients with high cardiovascular risk. Inclusion criteria include patients who either have a history of an ASCVD event with LDL-*C* > 70 mg/dL or are at high risk for an event with LDL-*C* > 90. The primary objective is to evaluate the efficacy of enlicitide chloride compared with placebo in increasing the time to the first occurrence of MACE including coronary heart disease death, ischemic stroke, MI, acute limb ischemia or major amputation or urgent arterial revascularization. The goal study enrollment is 14,550 patients with a significant proportion of high risk primary prevention patients.

#### 3.5.4 TRANSFORM

The TRANSFORM trial [79] utilizes an innovative approach to preventative care testing by assessing whether personalized care based on quantitative analysis and staging of coronary atherosclerosis reduces cardiovascular events compared with usual care. Instead of relying on traditional ASCVD risk factors, the study will utilize coronary CT angiography to stratify risk. The study enrolls patients without known symptoms of cardiovascular disease but who are at high risk for CVD due to risk factors such as diabetes, prediabetes and metabolic syndrome. The primary endpoint is total (first and subsequent) events for the composite endpoint of CV death, non-fatal MI, non-fatal ischemic stroke, non-fatal acute limb ischemia, clinically driven arterial revascularization, and hospitalization or urgent visit for heart failure.

#### 3.5.5 ACCLAIM-Lp(a)

Although several Lp(a) therapies are in development, at time of this publication, the only outcome trial to include high-risk primary prevention patients is ACCLAIM. The ACCLAIM-Lp(a) trial [80] is an ongoing phase III study evaluating the efficacy of lepodisiran, an siRNA designed to target the messenger RNA for the LPA gene, which plays a crucial role in the production of apolipoprotein(a), a key component of Lp(a) particles. Earlier trials demonstrated that a single dose of lepodisiran could reduce Lp(a) levels by up to 96 % within two weeks, with these reductions maintained at nearly a year in follow-up [81]. This study enrolls high-risk patients without a history of ASCVD, provided they have an Lp(a) level of ≥175 nmol/L and have 3 risk enhancers such as elevated CAC score, elevated hs-CRP, or diabetes. The primary endpoint is time to the first occurrence of cardiovascular death, nonfatal myocardial infarction, nonfatal stroke, or urgent coronary revascularization. Initiated in March 2024, the study is expected to conclude around March 2029.

## 4 Management of high-risk primary prevention patients

**Fig. 2:**
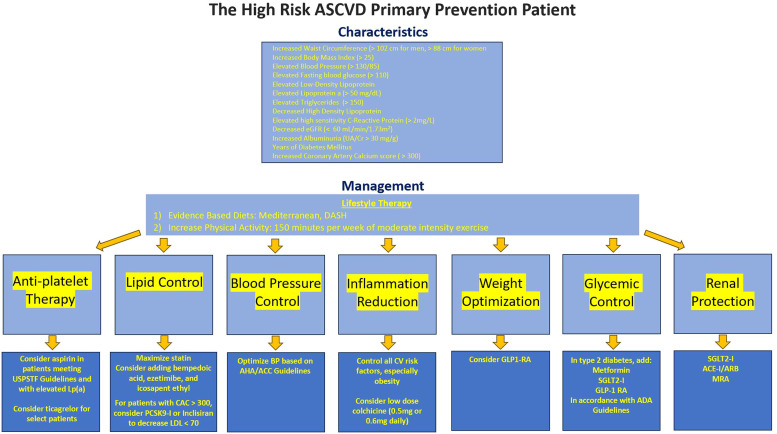
Characteristics and management of the high risk ASCVD primary prevention patient. TG = triglycerides, BP = blood pressure, eGFR = estimated glomerular filtration rate, cm = centimeter, hs-CRP = high sensitivity C-Reactive Protein, UA/Cr = urine albumin to creatinine ratio, CAC = coronary artery calcium, DM = diabetes mellitus, BMI = body mass index, LP(a) = lipoprotein a, mg = milligram, dL = deciliter, DASH = dietary approaches to stop hypertension, GLP-1 RA = Glucagon-like-peptide-1 receptor agonist, SGLT2-*I* = sodium glucose transporter 2 inhibitor, ACE-*I* = angiotensin-converting enzyme inhibitor, ARB = angiotensin receptor blocker, MRA = mineralocorticoid receptor antagonist, PCSK9-*I* = Proprotein convertase subtilisin/kexin type 9 inhibitor, AHA = American Heart Association, ACC = American College of Cardiology, ADA = American Diabetes Association, USPSTF = United States Preventive Services Task Force.

In this section, we review strategies for managing high risk primary prevention patients (Fig. 2).

### 4.1 Lifestyle modifications

As with all cardiovascular preventive strategies, management of lifestyle is the cornerstone of the management of high-risk primary prevention patients. A useful framework for conceptualizing the key modifiable factors and counseling patients is Life’s Essential 8 from the American Heart Association [82]. These components include a healthy diet (DASH or Mediterranean-style), physical activity (150 min of moderate or 75 min of vigorous physical activity per week), avoidance of nicotine exposure, 7–9 h of sleep per night, weight management (optimal body mass index of >18.5 and <25), optimal cholesterol, optimal blood glucose or hemoglobin A1c, and optimal blood pressure (<120/80 mmHg). Beyond maintaining a healthy lifestyle, therapeutic approaches for these and other key risk factors are discussed below.

### 4.2 Lipid management

The AHA Life’s Essential 8 defines optimal cholesterol as non-HDL-cholesterol <130 mg/dL (or <110 mg/dL on therapy) [82]. However, the concept of optimal lipid management is often complicated to explain to patients. Given that reducing LDL-C results in a reduction of risk regardless of baseline LDL-C, with an estimated relative risk reduction of 22 % per 39 mg/dL reduction in LDL-C [83], understanding baseline risk is more important than the absolute level of LDL-C, with the exception of very high levels, as emphasized by the most recent primary prevention guidelines [13]. Statin therapy is the first-line therapy for primary prevention. Adjuncts or alternatives to statin therapy include ezetimibe, bempedoic acid, icosapent ethyl and PCSK9 inhibitors. In particular, bempedoic acid has a demonstrated benefit in statin-intolerant patients, including high-risk primary prevention patients [57]. In patients with moderate hypertriglyceridemia, diabetes, and other cardiovascular risk factors, icosapent ethyl should also be considered for additional risk reduction [59]. PCSK9 inhibitors are associated with potent LDL-C lowering, and there are multiple ongoing trials of their use in primary prevention.

Generally, we recommend an LDL-C goal of < 70 for high-risk primary prevention ASCVD patients. In patients with CAC scoring, patients with moderate atherosclerosis or better (CAC < 300) should aim for LDL-*C* < 70 [84]. Patients with high burden (CAC > 300) should be considered secondary prevention patients and should be offered PCSK9 inhibitor or Inclisiran to target an LDL-*C* < 55 if not meeting goal on first-line therapy [84]. Treating to an LDL-C goal should especially be considered in patients with high CAC for age. Patients with familial hypercholesterolemia should be treated to at least an LDL-C goal of < 100, and providers should recommend lower goals in the setting of additional risk factors (< 70) or ASCVD (< 55) [85]. Patients with diabetes mellitus less than age 40 and CAC > 0 should be deemed high risk and counseled with shared decision making regarding initiation of high-intensity statin until they meet AHA/ACC class 1 recommendation guideline for high-intensity statin at age 40.

### 4.3 Blood pressure

Optimal blood pressure is defined as <120/80 mmHg, 120–129/<80 is elevated, 130–139/<90 is stage 1 hypertension, ≥140/90 is stage 2 hypertension [82]. Blood pressure management should follow existing guidelines. In particular, in primary prevention patients with 10-year ASCVD risk of ≥10 % and BP >130/80, pharmacologic therapy is recommended for a target of <130/80. Preferred first line agents are thiazide diuretics, angiotensin converting enzyme inhibitors, angiotensin receptor blockers, or calcium channel blockers. Non-pharmacological interventions are also important, including following a DASH style diet, weight loss if indicated, sodium restriction, increased physical activity, and moderating alcohol use [86].

### 4.4 Blood glucose

For patients with diabetes, use of agents with demonstrated cardiovascular benefits should be considered in high-risk primary prevention patients, particularly SGLT2 inhibitors and GLP-1 RA [87].

### 4.5 Antiplatelet therapy

Although aspirin use for primary prevention has progressively fallen out of favor and no longer has a strong recommendation in current guidelines [13,88], there may be a role for its use in certain high-risk primary prevention patients. In particular, there is evidence for a benefit from aspirin in patients with increased genetic risk for elevated Lp(a)[21,22] or elevated plasma levels of Lp(a) [23,24]. This benefit may be due to the overall increased cardiovascular risk in this population without available targeted therapies, and proposed pro-platelet and anti-fibrinolytic mechanisms associated with Lp(a) [89]. Although further studies with more robust evidence are needed, the most recent National Lipid Association statement on Lp(a) recommends discussing the risks and benefits of aspirin use with patients [20].

Another high-risk primary prevention population that may benefit from aspirin therapy includes individuals with CAC >100 without increased bleeding risk, based on prior studies simulating the potential risk and benefit of aspirin therapy [90,91].

### 4.6 Kidney disease

For patients with HTN and CKD with or without diabetes, renin-angiotensin-aldosterone inhibitors are first line agents. For patients with concomitant diabetes, SGLT-2 inhibitors and finerenone are recommended for prevention of progression of cardiovascular disease [46,47]. In CANVAS, approximately 34 % of the cohort was enrolled for primary prevention. While overall event rates were lower in the primary prevention cohort, canagliflozin reduced the primary endpoint compared to placebo hazard ratio 0.86; 95 % confidence interval 0.75–0.97; *P* < 0.001 for noninferiority, *P* = 0.02 [48]. Similar reductions in MACE were seen in the canagliflozin cohort in the CREDENCE trial [49]. The FIDELIO-DKD and FIGARO-DKD trials [46,92] demonstrated that finerenone significantly reduced renal and cardiovascular outcomes in patients with diabetes mellitus and a broad spectrum of CKD. The pooled analysis (FIDELITY) confirmed a cardiovascular benefit with finerenone irrespective of ASCVD history (primary endpoint occurrence 8.9 % in treatment group vs 9.7 % in placebo) [50].

Based on these data, in patients with albuminuria, diabetes, and hypertension, therapy with an angiotensin converting enzyme inhibitor or angiotensin receptor blocker is recommended [93]. In patients with diabetes, SGLT2 inhibitors, GLP-1 RA [93], or finerenone [46] should be considered.

### 4.7 Weight management

As noted above, GLP-1 RA are recommended in patients with diabetes and increased cardiovascular risk. These agents have the added benefit of weight loss. While these agents have not yet been studied in primary prevention patients without diabetes, the landmark SELECT trial [70] demonstrated a benefit in individuals with established cardiovascular disease and overweight/obesity without cardiovascular disease.

### 4.8 Inflammatory risk

Treatment of modifiable traditional cardiovascular risk factors, especially adopting the health habits of weight loss, exercise, and cessation of smoking can reduce serum CRP levels [26]. The landmark JUPITER trial demonstrated that, in primary prevention patients with elevated inflammatory risk (as evidenced by elevated hs-CRP levels), statin therapy was associated with a reduction in LDL-C, hs-CRP and cardiovascular events [55]. As such, statins are first-line for management of risk in these patients. After the LoDoCo2 trial [71] demonstrated a benefit to colchicine daily in patients with chronic coronary artery disease, colchicine was approved for individuals with established ASCVD or with multiple risk factors for ASCVD. The efficacy of colchicine in reducing ASCVD events relative to baseline hs-CRP level has not been studied in a prospective trial and remains an important unanswered clinical question. Based on retrospective data and expert opinion only, providers may consider measuring hs-CRP before initiating colchicine and more strongly consider this therapy in patients with hs-CRP ≥ 2 mg/L [2,25].

In patients with rheumatologic disorders, consultation with a rheumatologist for optimal control of systemic inflammation with biologic therapies recommended for the rheumatologic disorder is also prudent for ASCVD risk reduction.

## 5 Conclusions

In 1895, Poet Joseph Malins penned the poem “The Fence or The Ambulance”[94] which presciently describes 21st century healthcare delivery, nearly 130 years later. Current healthcare paradigms deliver highly skilled care and large resources to target acute events, and for patients who survive, provide ongoing expensive secondary prevention care. Malins describes a situation of people living by a cliff, where “a duke and full many a peasant” have fallen. A reactionary approach deploying an ambulance “in the valley” below the cliff rather than place “a fence ‘round the edge of the cliff” was chosen as a “fence may be useful or not, it is true, but each heart became full of pity for those who slipped over the dangerous cliff.”

The ambulance approach requires an intensive, active approach where many individuals may not survive their fall despite an enormous amount of resource and time. The placement of a low-maintenance fence may not stop all falls, and in some cases may not be required at all, but it may provide broad and enduring protection to the community that is superior to the ambulance for those who never fall.

ASCVD development is a complex process with development of disease well before events manifest. A primordial risk reduction approach leverages assessing biomarkers for risk and utilizing stratification tools to assess each patient as to their proximity to “the cliff", their cliff’s steepness and height, and the ease in which one may slip and fall from the edge. Therapeutic “fences” may not need to be used at all in some patients. However, in the high-risk individual, earlier rather than later deployment is preferred and may be much more effective (Fig. 1). We describe concomitant therapeutic aims in parallel that build the fence for the foundation of lifelong optimal lifestyle therapy (Fig. 2).

Currently, American and international guidelines use risk scores that one can calculate only when individuals have reached the age of 30 or 40. However, in the presence of abnormal biomarkers, genetic factors such as Familial Hypercholesterolemia, and other disease states, e.g., diabetes type I and II, ASCVD can be recognized earlier than the age at which these calculators have been validated to stratify risk. The standard approach using risk calculators does not prompt patients and clinicians to evaluate, discuss, and implement therapies for primordial risk reduction.

The evidence for cardiovascular benefit in initiating primordial preventive therapies is clear and continues to grow from foundational statin trials such as JUPITER. Table 1 provides a list of other non-statin lipid lowering and other biomarker targeting therapies that demonstrate incremental risk reduction in contemporary cardiovascular outcome trials. Finally, there are ongoing studies focused on reducing cardiovascular risk in primary prevention populations not only by targeting lipids, but also other risk components (i.e., renal dysfunction, elevated blood pressure, overweight, and inflammation). Globally, nearly one in three men and women fall off the cliff. While we work on recovering them in the ambulance, we have opportunities to identify early who are the next that may fall, and build the fences to avoid mortality and morbidity and focus our resources to improve quality and quantity of life.

## Funding support statement

Dr. Bhatia is supported by National Institutes of Health, Grant 1K08HL166962. A grant from the Larry Hillblom Foundation supports Dr. Taub. Esperion financially supported the development of Fig. 1 in the manuscript.

## CRediT authorship contribution statement

**Michael Ahlers:** Writing – review & editing, Writing – original draft. **Sonya John:** Writing – review & editing, Writing – original draft. **Harpreet Bhatia:** Writing – review & editing. **Viet Le:** Writing – original draft. **Pam Taub:** Writing – review & editing, Conceptualization.

## Declaration of competing interest

The authors declare the following financial interests/personal relationships which may be considered as potential competing interests:

Pam Taub reports financial support was provided by Esperion Therapeutics Inc. Pam Taub reports a relationship with Novartis Pharmaceuticals Corporation that includes: consulting or advisory. Pam Taub reports a relationship with Esperion Therapeutics Inc that includes: consulting or advisory. Pam Taub reports a relationship with Amarin Pharma Inc that includes: consulting or advisory. Pam Taub reports a relationship with Amgen Inc that includes: consulting or advisory. Pam Taub reports a relationship with Novo Nordisk Inc that includes: consulting or advisory. Pam Taub reports a relationship with Medtronic that includes: consulting or advisory. Pam Taub reports a relationship with Edwards that includes: consulting or advisory. Pam Taub reports a relationship with Boehringer Ingelheim Corp USA that includes: consulting or advisory. Pam Taub reports a relationship with Jazz Pharmaceuticals Inc that includes: consulting or advisory. Pam Taub reports a relationship with Milestone Pharmaceuticals that includes: consulting or advisory. Pam Taub reports a relationship with Bayer Corporation that includes: consulting or advisory. Pam Taub reports a relationship with Eli Lilly and Company that includes: consulting or advisory. Viet Le reports a relationship with Novartis Pharmaceuticals Corporation that includes: consulting or advisory. Viet Le reports a relationship with Amarin Pharma Inc that includes: consulting or advisory. Viet Le reports a relationship with Pfizer that includes: consulting or advisory. Viet Le reports a relationship with Lexicon Pharmaceuticals Inc that includes: consulting or advisory. Viet Le reports a relationship with Idorsia Pharmaceuticals Ltd that includes: consulting or advisory. Harpreet Bhatia reports a relationship with Kaneka Corporation that includes: consulting or advisory. Harpreet Bhatia reports a relationship with Novartis Pharmaceuticals Corporation that includes: consulting or advisory. Harpreet Bhatia reports a relationship with Arrowhead Pharmaceuticals Inc that includes: consulting or advisory. Harpeet Bhatia reports a relationship with Abbott that includes: consulting or advisory. If there are other authors, they declare that they have no known competing financial interests or personal relationships that could have appeared to influence the work reported in this paper.
